# Clinical Evaluation and Management of Overlap Syndrome (OS) and Obesity Hypoventilation Syndrome (OHS)

**DOI:** 10.3390/clockssleep4040055

**Published:** 2022-12-06

**Authors:** Pasquale Tondo, Giulia Scioscia, Anela Hoxhallari, Roberto Sabato, Simone Sorangelo, Giuseppe Mansueto, Antonella Giuliani, Maria Pia Foschino Barbaro, Donato Lacedonia

**Affiliations:** 1 Department of Medical and Surgical Sciences, University of Foggia, 71122 Foggia, Italy; 2 Department of Specialistic Medicine, Respiratory and Intermediate Care Unit, Policlinico Foggia University Hospital, 71122 Foggia, Italy

**Keywords:** COPD, management, OHS, overlap syndrome, sleep apnea, treatment

## Abstract

**Background and Aim:** Sleep-disordered breathing (SDB) is an extremely common disorder with a high impact on morbidity and mortality. The purpose of this study was to compare overlap syndrome (OS) and obesity hypoventilation syndrome (OHS) and to highlight and understand the differences between them. **Material and Methods:** The study was conducted retrospectively on 132 subjects selected by consecutive sampling from those attending our unit for suspected SDB. After clinical evaluation as well as functional and sleep investigations, the population was divided according to diagnosis in OS and OHS; then, the clinical parameters of two groups were compared with different statistical analysis. **Results:** The subjects with OHS were younger and reported higher rated daytime sleepiness (*p* = 0.005). In addition, they presented more nocturnal respiratory events (apnea-hypopnea index (AHI) 63.61 ± 22.79 events·h^−1^ vs. AHI_OS_ 42.21 ± 22.91 events·h^−1^, *p* < 0.0001) at the sleep investigation as worse gas exchange during sleep leading to a higher percentage of nocturnal hypoxemia (*p* < 0.0001). In contrast, subjects with OS had more an impaired respiratory function. With regard to night-time ventilatory therapy, more subjects with OS were effectively treated with continuous positive airway pressure (CPAP) (*p* = 0.011), while more OHS were treated with auto-adjusting PAP (APAP) (14% vs. 1%, *p* = 0.008). **Conclusions:** The present study tried to establish a framework for OS and OHS because proper management of the two disorders would reduce their burden on healthcare.

## 1. Introduction

Sleep-disordered breathing (SBD) is an extremely common disease that afflicts approximately one billion individuals between the ages of 39 and 70 [1]. Among the various forms of SBD, the most common is obstructive sleep apnea (OSA) characterised by repetitive episodes of complete or partial upper airway collapse and associated with intermittent nocturnal hypoxia [2]. In addition, craniofacial abnormalities are widely recognised among the causes of SDB [3,4].

In general, the alterations of gas exchanges during sleep cause an increased risk of a large number of diseases [5]. Several studies have demonstrated a directly proportional relationship between the number of cardiovascular risk factors, such as the development of arrhythmias, and OSA severity [6,7,8]. In addition, the research demonstrated a close association of SDB with endocrinological and neurological diseases, psychiatric disorders but also malignancies [9,10,11,12], with an associated increased risk of mortality rate [13].

When obstructive respiratory diseases, such as chronic obstructive pulmonary disease (COPD) are associated with OSA (Overlap Syndrome (OS)), the mortality risk is extremely increased. Indeed, the presence of both conditions, OSA and COPD, causes an increase in systemic inflammation and oxidative stress that contributes to the development of cardiovascular disease [14,15].

Another respiratory condition with a major impact on health is obesity-hypoventilation syndrome (OHS) [16]. This condition, established by clinical evaluation, blood gas analysis and a sleep investigation, is characterised by diurnal hypercapnia linked to various mechanisms, such as obesity and alterations in the respiratory drive and mainly causes metabolic and cardiovascular disorders [17].

Given the health impact and consequences of these SBD, rapid initiation of therapeutic treatment is crucial. In general, the first-line treatment is continuous positive airway pressure (CPAP) [18]. As these two respiratory conditions are complex, a proportion of patients do not correct all nocturnal respiratory events with CPAP or do not tolerate the pressures delivered, and, therefore, it is necessary to choose alternative ventilatory modalities that are effective in these patient classes.

For this reason, the present study aims to evaluate both the respiratory profiles and the management of the two conditions, OS and OHS, with attention also paid to the choice of the appropriate ventilatory device and, thus, better understand the differences in these two SBD.

## 2. Results

The characteristics of the sample studied are summarised in Table 1.

Specifically, of this sample 105 had experience of OS and 27 of OHS with an overall mean AHI of 46.21 ± 24.29 events·h^−1^, oxygen desaturation index (ODI) of 45.96 ± 27.06 events·h^−1^ and NH in 47% of cases.

**Figure 1:**
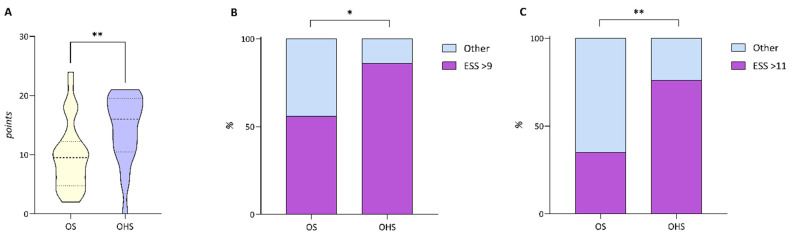
The violin plot (**A**) represents the global mean Epworth Sleepiness Scale (ESS) score, while bar charts (**B**,**C**) the differences between overlap syndrome (OS) and obesity hypoventilation syndrome (OHS) at several thresholds of ESS. * *p* < 0.05 ** *p* < 0.001.

Dividing the population according to the two disorders, OS and OHS, we noted that subjects with OHS are younger and obviously obese (Table 2). Furthermore, subjects with OHS reported greater daytime sleepiness assessed by the Epworth Sleepiness Scale (ESS) for both the overall mean ESS (*p* = 0.005) and the two thresholds used as discriminating values for daytime sleepiness (ESS > 9 points, *p* = 0.022 and ESS > 11 points, *p* = 0.003), as shown in Figure 1.

**Figure 2:**
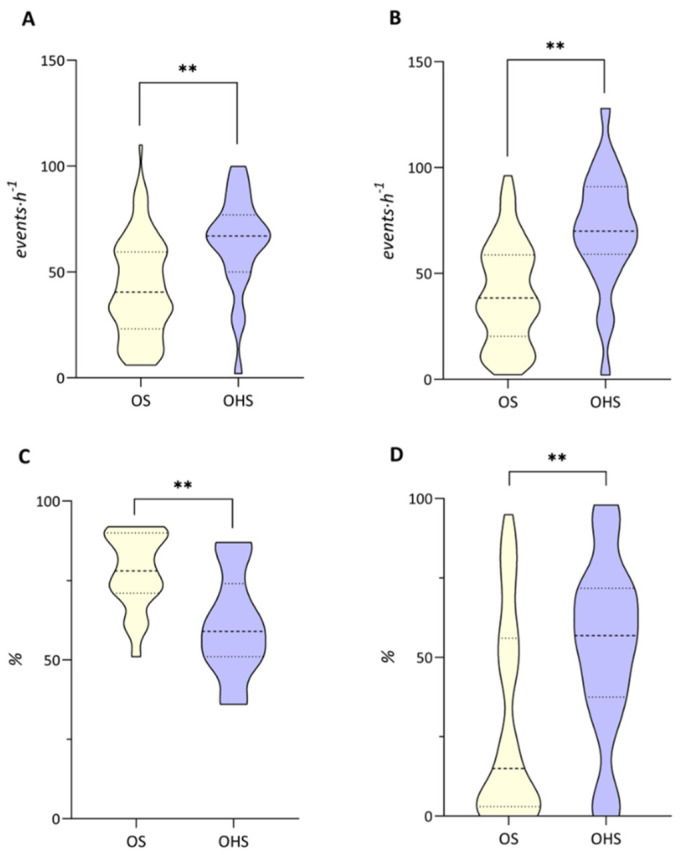
Comparison of sleep parameters between overlap syndrome (OS) and obesity hypoventilation syndrome (OHS): (**A**) apnea-hypopnea index (AHI), (**B**) oxygen desaturation index (ODI), (**C**) oxygen saturation (SaO_2_) and (**D**) time in bed with SaO_2_ <90% (T90). ** *p* < 0.001.

Assessing the sleep investigation parameters, we observed that subjects with OHS had more nocturnal respiratory events (AHI 63.61 ± 22.79 events·h^−1^ vs. AHI_OS_ 42.21 ± 22.91 events·h^−1^, *p* < 0.0001) and a greater number of desaturations (ODI 70.26 ± 27.87 events·h^−1^ vs. ODI_OS_ 40.37 ± 23.69 events·h^−1^, *p* < 0.0001) and, consequently, a greater number of subjects with severe OSA (91% vs. 70% OS, *p* = 0.036) (Figure 2). In addition, subjects with OHS had a lower SaO_2_ (*p* = 0.001) and a higher percentage of NH (*p* < 0.0001).

Conversely, when assessing respiratory function, subjects with OS had a lower FEV_1_ (*p* < 0.0001) than the OHS.

Of the whole sample, we assessed the ventilatory modes used, observing that more subjects with OS were treated with CPAP vs. the OHS (*p* = 0.011), although we did not observe differences in the overall mean CPAP pressure and the various CPAP-pressure thresholds taken into account (CPAP pressure > 8 cmH_2_O, >10 cmH_2_O, >12 cmH_2_O) to cluster the subjects.

In CPAP-unresponsive subjects, it was noted that the APAP mode was used more for OHS (14% vs. 1% of OS, *p* = 0.008) while there was no difference for bilevel PAP (BPAP). In both groups, oxygen supplementation was added to the ventilator in the same percentage of the population as well as adherence to ventilatory therapy at 90-days was ~90%.

## 3. Discussion

The present study retrospectively analysed the general characteristics found between the two SDBs under consideration, i.e., OS and OHS, from the differences in the underlying pathophysiological mechanisms to the therapeutic management assessed in our Unit. To the best of our knowledge, there are no other comparison studies in the literature that have evaluated the management and characteristics of the two diseases.

The first difference we observed from our case series was the older age of the subjects with OS. Some studies found that OS patients were older and less obese than OSA controls [19]. This result is not of unequivocal interpretation. Regardless, COPD has two clinical phenotypes the ‘pink puffer’ and the ‘blue bloater’; the former characterised by low BMI and the presence of emphysema that has been shown to have less likelihood of OSA, and the latter with higher BMI seems to be predisposed to the development of OSA [14,20,21]. On the other hand, smoking, in addition to being a recognised risk factor for COPD, is also a risk factor for OSA, mainly through two mechanisms: firstly, increased sleep instability linked to the ‘rebound effect’ of nicotine in which the effects that favour increased upper airway tone are reversed during nocturnal nicotine withdrawal; secondly, smoking-related airway inflammation [22].

In general, in patients with COPD physiological changes during sleep cause alterations in gas exchanges. This phenomenon is mainly evident during the REM phase because of the muscular atony characteristic of this phase. In addition, hyperinflation worsens this condition because it reduces diaphragmatic contraction resulting in a compensatory mechanism through the use of accessory muscles suitable for maintaining an effective ventilation. This compensatory mechanism over time leads to work of breathing (WOB) with slow depletion of muscle strength. All this contributes to nocturnal hypoventilation and subsequent hypoxemia [23]. Therefore, this slow functional decline or weight gain over the years could explain a late onset of the overlap between the two diseases. On the other hand, the diagnosis of OHS is typically made during the fifth and sixth decades of life, an earlier age compared to patients with OS, taking into account that it is generally diagnosed when the patient reaches a high state of acuity in the form of acute or chronic hypercapnic respiratory failure. In addition, 75% are misdiagnosed and treated for obstructive lung disease (most commonly chronic obstructive pulmonary disease) even without evidence of obstruction in respiratory function tests [17].

Another difference we observed in our sample was the reported daytime sleepiness; in fact, patients with OHS reported greater daytime sleepiness even at different ESS thresholds. Among the main factors recognized as underlying daytime sleepiness was nocturnal hypoxia. This phenomenon is also more pronounced in patients with OHS compared to patients with OSA alone; they have higher arterial oxygen tension <90%, lower mean saturation and longer desaturation [24,25].

This result also seems to be confirmed in our sample with OHS compared with OS. In fact, almost twice as many OHS manifested nocturnal hypoxemia (NH) as the group with OS. The same result was observed for the other polysomnographic indices. In fact, patients with OHS had worse sleep alterations, both in terms of severity calculated by AHI and gas exchange.

The pathophysiological traits underlying the two diseases are different. OHS is related to three main mechanisms: (i) obesity that alters respiratory function, (ii) alterations in respiratory drive, and (iii) respiratory changes during sleep [17]. These phenomena characterise individuals with OHS who may present two distinct phenotypes: first, the predominant OSA phenotype thus characterised by more obstructive events during sleep; second, the hypoventilation phenotype thus characterised by hypoventilation during sleep defined according to the AASM also as an increase in PaCO_2_ > 10 mmHg compared with a supine awake value [26].

In contrast, OS-related mechanisms include altered respiratory drive, increased airway resistance, altered lung mechanics and respiratory muscles resulting in ineffective alveolar gas exchange. As previously mentioned, the main abnormalities during sleep occur mainly in the REM phase when, associated with lower muscle activity, minute ventilation is reduced by up to ~30% compared with wakefulness, resulting in a major decrease in minute ventilation in patients with COPD.

In addition, muscle atony during the REM phase of sleep results in reduced functional residual capacity (FRC) and nocturnal hypoxia causing further reduction in minute ventilation for rapid and shallow breathing in response to these phenomena [27].

These ongoing mechanisms during the night are also reflected in the daytime status with a reduction in PaO_2_ [28] and functional decline [29].

Therefore, the underlying pathophysiological differences in these two diseases may explain the results found in our study, that is, a lower forced expiratory volume in the first second (FEV_1_) in patients with OS and a higher PaCO_2_ in patients with OHS.

Regarding comorbidities, hypertension is the disease most frequently associated with OS and OHS followed by cardiovascular events and diabetes mellitus with a higher prevalence in OHS patients as also confirmed by the study of Lacedonia et al. [30].

Finally, when we analysed the ventilatory therapy, we noticed that almost all of the OS group (~90%) were effectively treated with CPAP, while CPAP was effective to a lesser extent (~70%) in the OHS group. The former finding is supported by several studies that have confirmed that CPAP is the standard treatment for OS [31].

Conversely, with regard to OHS, Masa et al. compared the efficacy of non-invasive ventilation (NIV) with CPAP. In this recent review, the pure hypoventilation phenotype of OHS seems to respond better to NIV, in contrast, the phenotype with greater obstructive events during sleep seems to respond more to CPAP. However, the authors recommend that patients on CPAP treatment be closely monitored so that if there is a lack of response to treatment, understood as failure to improve gas exchange and, thus, global respiratory failure, a therapeutic switch to NIV can be initiated [17]. That is also because scientific evidence reports the efficiency of night ventilation in reducing mortality rates [32]. In fact, survival rates in patients with OHS undergoing overnight ventilation are ~97–70% within 1–5 years and, thus, higher than untreated OHS [33].

A recent systematic review suggests that both ventilatory modalities are useful in improving gas exchange, sleep quality and, consequently, daytime sleepiness and quality of life. However, the authors recommend using CPAP as first-line therapy because it requires fewer resources from NIV [34].

Among the various ventilatory modalities, we found in our sample that 14% of patients with OHS responded to night-time therapy with APAP. We found no other evidence to support this result other than related to the use of an auto-trilevel [35] or in the use of APAP for titration [36]. The evidence in favour of using different levels of auto-adjusting PAP in OSA is plenty [18,37,38]. Perhaps the use of APAP without a large ∆ pressure could, on the one hand, clean up nocturnal obstructive events and, on the other hand, improve ventilation, but studies are needed to support this hypothesis.

## 4. Materials and Methods

A retrospective, observational study was conducted on consecutive subjects who referred to our unit for suspected SDB between 2017–2020.

All participants performed a visit for clinical examination, pulmonary function testing (PFT) and blood gas analysis. Demographic, anthropometric and clinical data were collected at clinical examination. Subsequently, participants performed cardiorespiratory monitoring (CRM).

After these examinations, the participants were included in the study if diagnosed with OS (presence of OSA [apnea-hypopnea index (AHI) >5 events·h^−1^] overlapped with COPD [post-bronchodilator FEV1·FVC^−1^ ratio <70% at PFT [39]] or diagnosis of OHS (BMI > 30 kg·m^−2^, daytime alveolar hypoventilation with PaCO_2_ > 45 mmHg in the absence of other possible causes of hypoventilation) [16].

Exclusion criteria: medical history of heart failure or neuromuscular disease.

### 4.1. Sleep Protocol

All participants performed a CRM (polysomnography type III, Alice PDx, Philip Respironics, Murrysville, PA, USA) that recorded the following signals during the night: airflow with nasal cannulas and thermistors, impedance of the respiratory effort in the chest and abdomen, body position, SaO_2_ and heart rate (HR). The sleep investigations were manually scored by a sleep expert physician; hypopneas were classified in the presence of a decrease in airflow ≥ 30% compared to baseline, lasting at least 10 s and associated with ≥3% oxygen desaturation. The classification of respiratory events (obstructive, central or mixed) was performed according to the AASM criteria [40].

Participants were subsequently started on CPAP titration trials according to the AASM recommendations using an auto-adjusting positive airway pressure (APAP) device (ICON, Fisher & Paykel (East Tamaki, Auckland, New Zealand) or S9-Autoset, ResMed (San Diego, CA, USA)) to set the therapeutic pressure [18,41]. After five nights in APAP mode, the therapeutic CPAP pressure was set and tested by CRM. In the case of patients unresponsive or non-tolerant to CPAP, other ventilatory modes were used. In addition, in the case of patients with nocturnal hypoxemia (NH), defined as total time in bed with SaO_2_ < 90% (T90) was ≥30%, despite optimal ventilatory treatment an oxygen supplement was added.

### 4.2. Pulmonary Function Test

PFTs were performed in our unit’s respiratory pathophysiology laboratory using a spirometer (Sensormedics, Yorba Linda, CA, USA). The spirometer was calibrated daily using a 3-litre syringe. Basal examination was calculated as a percentage of the expected normal value of the best of three reproducible spirometric values, while post spirometry was performed 15 min after the subject had inhaled 400 mg salbutamol.

Accordingly, participants with a post-bronchodilator FEV_1_·FVC^−1^ < 70% were classified as COPD according to the GOLD guidelines [35] and included in the study, while participants with a post-bronchodilator FEV_1_ increase of 12% were excluded.

### 4.3. Blood Gas Analysis

The ABG was performed to analyse respiratory gas exchange during room air breathing. The analysis was plotted with the patient in a sitting position on the day following the CRM recording within 1 h after awakening. PaO_2_, PaCO_2_, HCO_3_^−^ and pH were measured using a blood gas analyser (GEM^®^Premier™4000, Birchwood, UK).

### 4.4. Statistical Analysis

The sample distribution was assessed by means of the Shapiro-Wilk test. Continuous variables were expressed as mean ± standard deviation, while categorical variables were expressed as percentages.

The population was divided into two groups according to the diagnosis: OS (COPD plus OSA) and OHS and then, the two groups were compared using either the *t*-test or the Mann-Whitney U-test, as appropriate.

A *p*-value < 0.05 was considered statistically significant. IBM SPSS Statistics (version 26, IBM Corp., Chicago, IL, USA) and GraphPad (version 8, GraphPad Software Inc., La Jolla, CA, USA) softwares were used for analyses and graphs, respectively.

## 5. Conclusions

In conclusion, OS and OHS are two extremely complex and high-impact nocturnal respiratory disorders. Taking into account their distinctive pathophysiological features, an appropriate clinical approach would enable an earlier and effective targeted management of the disease. Therefore, further studies that also evaluate biomarkers and ventilatory modalities other than those described in our work are needed to reduce the health impact of these two disorders.

## Figures and Tables

**Table 1:** General characteristics of population.

Variables	Total
	*n* = 132
**Sex, male, %**	77%
**Age, years**	64.4 ± 11.63
**BMI, kg·m^−2^**	33.65 ± 7.67
**Neck, cm**	45.76 ± 4.13
**Smoking habits, %**	27%
**Comorbidities**	
**CVD, %**	39%
**Hypertension, %**	70%
**Cerebrovascular, %**	9%
**Endocrinological disorders-non-diabetes, %**	12%
**Diabetes, %**	27%
**Daytime sleepiness**	
**ESS score, points**	11.51 ± 6.08
**ESS ≥ 9 points, %**	67%
**ESS ≥ 11 points, %**	51%
**Sleep data**	
**AHI, events·h^−1^**	46.21 ± 24.29
**Severe OSA, %**	74%
**ODI, events·h^−1^**	45.96 ± 27.06
**Minimum SaO_2_, %**	71.26 ± 15.91
**T90, %**	34.39 ± 31.61
**NH (T90 > 30%), %**	47%
**Respiratory and performance status**	
**FVC, % predicted**	84.76 ± 17.26
**FEV_1_, % predicted**	70.33 ± 19.33
**FEV_1_·FVC^−1^**	63.73 ± 12.68
**pH**	7.42 ± 0.03
**PaO_2_, mmHg**	70.66 ± 10.47
**PaCO_2_, mmHg**	41.73 ± 5.93
**SaO2, %**	94.12 ± 2.74
**HCO_3_^−^, mmol·l^−1^**	25.84 ± 3.23
**6MWT, mt**	295.29 ± 112.35
**Night-time therapy**	
**Reject PAP, %**	1%
**CPAP, %**	88%
**CPAP Pressure, cmH_2_O**	11.01 ± 1.92
**CPAP ≥ 8 cmH_2_O, %**	97%
**CPAP ≥ 10 cmH_2_O, %**	83%
**CPAP ≥ 12 cmH_2_O, %**	31%
**BPAP, %**	7%
**APAP, %**	4%
**PAP compliance, %**	90%
**O_2_ supplement, %**	29%

*Abbreviations*: 6MWT = 6 min walking test; APAP = auto-adjusting positive airway pressure; AHI = apnea-hypopnea index; BMI = body mass index; BPAP = bilevel positive airway pressure; CPAP = continuous positive airway pressure; CVD = cardiovascular disease; ESS = Epworth sleepiness scale; FEV_1_ = forced expiratory volume in 1 s; FVC = forced vital capacity; HCO_3_^−^ = bicarbonates; NH = nocturnal hypoxemia; ODI = oxygen desaturation index; PAP = positive airway pressure; SaO_2_ = oxygen saturation; T90 = total sleep time with SaO_2_ < 90%.

**Table 2:** Comparison between patients with Overlap Syndrome (OS) and patients with Obesity Hypoventilation Syndrome (OHS).

Variables	OS	OHS	*p*
	*n* = 105	*n* = 27	
**Sex, male, %**	80%	63%	0.063
**Age, years**	66.16 ± 10.87	57.32 ± 12.11	0.001
**BMI, kg·m^−2^**	32.17 ± 6.74	39.06 ± 8.54	<0.001
**Neck, cm**	45.78 ± 3.85	45.72 ± 4.6	0.965
**Smoking habits, %**	23%	41%	0.098
**Comorbidities**			
**CVD, %**	37%	48%	0.3
**Hypertension, %**	69%	74%	0.582
**Cerebrovascular, %**	10%	4%	0.278
**Endocrinological disorders-non-diabetes, %**	12%	11%	0.858
**Diabetes, %**	24%	41%	0.079
**Daytime sleepiness**			
**ESS score, points**	9.74 ± 5.7	14.38 ± 5.69	0.005
**ESS ≥ 9 points, %**	56%	86%	0.022
**ESS ≥ 11 points, %**	35%	76%	0.003
**Sleep data**			
**AHI, events·h^−1^**	42.21 ± 22.91	63.61 ± 22.79	<0.001
**Severe OSA, %**	70%	91%	0.036
**ODI, events·h^−1^**	40.37 ± 23.69	70.26 ± 27.87	<0.001
**Minimum SaO_2_, %**	77.95 ± 11.76	61 ± 16.28	0.001
**T90, %**	30.23 ± 31.05	53.09 ± 27.6	0.002
**NH (T90 > 30%), %**	39%	82%	<0.0001
**Respiratory and performance status**			
**FVC, % predicted**	85.4 ± 17.74	82.2 ± 15.28	0.409
**FEV1, % predicted**	67.39 ± 18.45	82.32 ± 18.5	<0.001
**FEV1·FVC^−1^**	59.49 ± 10.03	80.88 ± 5.98	<0.001
**pH**	7.42 ± 0.03	7.4 ± 0.03	<0.001
**PaO_2_, mmHg**	71.07 ± 10.97	69.1 ± 8.27	0.385
**PaCO_2_, mmHg**	39.99 ± 4.87	48.48 ± 4.76	<0.001
**SaO_2_, %**	94.16 ± 2.92	93.94 ± 1.9	0.709
**HCO_3_^−^, mmol·l^−1^**	25.05 ± 2.89	28.89 ± 2.65	<0.001
**6MWT. mt**	294.75 ± 110.97	299.86 ± 133.04	0.91
**Night-time therapy**			
**Reject PAP, %**	0%	5%	0.058
**CPAP, %**	92%	71%	0.011
**CPAP Pressure, cmH_2_O**	10.95 ± 2.05	11.29 ± 1.13	0.579
**CPAP ≥ 8 cmH_2_O, %**	97%	100%	0.524
**CPAP ≥ 10 cmH_2_O, %**	80%	100%	0.089
**CPAP ≥ 12 cmH_2_O, %**	32%	25%	0.629
**BPAP, %**	7%	10%	0.66
**APAP, %**	1%	14%	0.008
**PAP compliance, %**	90%	89%	0.977
**O_2_ supplement, %**	26%	50%	0.091

*Abbreviations:* 6MWT = 6 min walking test; APAP = auto-adjusting positive airway pressure; AHI = apnea-hypopnea index; BMI = body mass index; BPAP = bilevel positive airway pressure; CPAP = continuous positive airway pressure; CVD = cardiovascular disease; ESS = Epworth sleepiness scale; FEV_1_ = forced expiratory volume in 1 s; FVC = forced vital capacity; HCO_3_^−^ = bicarbonates; NH = nocturnal hypoxemia; ODI = oxygen desaturation index; PAP = positive airway pressure; SaO_2_ = oxygen saturation; T90 = total sleep time with SaO_2_ < 90%.

## Data Availability

The data presented in this study are available on request from the corresponding author.
